# Associations of Wearable Activity Tracker Use With Physical Activity and Health Outcomes in Patients With Cancer: Findings from a Population-Based Survey Study

**DOI:** 10.2196/51291

**Published:** 2024-10-22

**Authors:** Weijiao Zhou, Shaomei Shang, Youmin Cho

**Affiliations:** 1 School of Nursing Peking University Beijing China; 2 College of Nursing Chungnam National University Daejeon Republic of Korea

**Keywords:** physical activity, exercise, wearable tracker, wearable device, cancer, oncology, cancer survivorship, wearable, wearables, tracker, trackers, health outcome, health outcomes, HINTS, survey, surveys, national, bivariate, epidemiology, epidemiological, association, associations

## Abstract

**Background:**

Physical inactivity is a global issue for cancer survivors. Wearable activity trackers are promising to address physical inactivity by providing real-time feedback on physical activity and offering opportunities for self-monitoring and goal setting. Meta-analysis has reported the effects of interventions that incorporate wearable activity trackers on improved physical inactivity and related health outcomes (eg, BMI, anxiety and depression, and self-rated health status). However, wearable activity trackers were often used as an adjunct to physical activity interventions, and the effectiveness of wearable activity trackers alone is unknown.

**Objective:**

This study aims to determine the association of wearable activity trackers with physical activity and health outcomes in patients with cancer.

**Methods:**

Data from 957 cancer survivors from the Health Information National Trends Survey–Surveillance, Epidemiology, and End Results (HINTS-SEER) were analyzed. The outcome variables examined were time spent in moderate to vigorous physical activity, weekly frequency of strength training, BMI, anxiety and depression levels, and self-assessed health status. The primary independent variable was whether cancer survivors had used wearable activity trackers within the past 12 months. Design-based linear regression for continuous outcome variables and ordinal logistic regression for ordinal outcome variables were conducted to determine the associations after controlling for sociodemographic, cancer-related, and health-related factors. All data analyses accounted for the complex survey design and sample weights.

**Results:**

Only 29% of cancer survivors reported wearable activity tracker use. Bivariate analyses showed that younger age (*P*<.001), higher education (*P*=.04), higher income (*P*<.001), and an employed status (*P*<.001) were significantly associated with wearable activity tracker use. Wearable activity tracker use was significantly associated with higher time spent in moderate to vigorous physical activity (adjusted =37.94, 95% CI 8.38-67.5; *P*=.01), more frequent strength training per week (adjusted odds ratio [OR] 1.50, 95% CI 1.09-2.06; *P*=.01), and better self-rated health status (adjusted OR 1.58, 95% CI 1.09-2.29; *P*=.01), but not with BMI or anxiety and depression.

**Conclusions:**

This study suggests that the uptake of wearable activity trackers is low and highlights the digital divide among patients with cancer. This study has confirmed the associations of wearable activity tracker use with physical activity and self-rated health, supporting using wearable activity trackers as a promising tool to facilitate physical activity promotion.

## Introduction

Physical activity improves health outcomes of patients with cancer, including cardiovascular fitness, muscle strength, cancer-related fatigue, health-related quality of life, and depression [1-3]. Physical activity guidelines for cancer survivors recommend at least 150 minutes of moderate physical activity per week [4]. However, only a minority of cancer survivors meet the physical activity recommendations [5,6]. Physical inactivity is a global issue for cancer survivors, and much work remains to be done to improve physical activity [7].

Wearable activity trackers offer an appealing, low-cost tool to address physical inactivity and, therefore, improve related health outcomes (eg, physiological outcomes, psychological outcomes, and self-rated health status). These devices provide real-time feedback on physical activity and offer opportunities for self-monitoring and goal setting. A review of systematic reviews [8] has reported that interventions that incorporate wearable activity trackers improve moderate to vigorous physical activity with an average effect size of 0.3 (6 minutes per day); an increase of 5-10 minutes per day of moderate to vigorous physical activity is considered clinically significant [9]. A meta-analysis of cancer survivors has found that wearable activity trackers improve moderate to vigorous physical activity with an effect size of 0.6 [10]. In addition, using wearable activity trackers has been shown to be associated with improved physiological outcomes (eg, BMI) [8], and it has the potential to improve psychological outcomes (eg, anxiety and depression) [8], and achieve positive self-rated health [11]. However, wearable activity trackers were often used as an adjunct to physical activity intervention or weight loss programs, which limits the ability to rule out the influence of other physical activity intervention components and determine the effectiveness of wearable activity trackers alone.

A few observational studies have examined the associations of wearable activity trackers with physical activity among general adults [12] and older adults [13], but the generalization to patients with cancer is limited. To address this gap, this study aimed to determine the association of wearable activity trackers with physical activity (moderate to vigorous physical activity and strength training) and health outcomes (BMI, anxiety and depression, and self-rated health status) in patients with cancer, using a population-based survey dataset obtained from 3 large cancer registries.

## Methods

### Data Sources and Study Population

We analyzed the Health Information National Trends Survey–Surveillance, Epidemiology, and End Results (HINTS-SEER) dataset. The Health Information National Trends Survey (HINTS) is a nationally representative survey that examined the knowledge, attitudes, and usage of cancer- and health-related information by American adults [14]. The initial HINTS encompassed both adults with and those without cancer, whereas the HINTS-SEER focused on a larger sample of adults with cancer from 3 SEER cancer registries in Iowa, New Mexico, and the Greater Bay Area of California, excluding individuals with only non–melanoma skin cancer diagnoses [15]. The data collection for HINTS-SEER took place by mail between January and August 2021. Among 1234 cancer survivors in the final HINTS-SEER sample, 277 respondents were excluded (21 had missing information about wearable activity use, 98 had missing information about outcome variables, and 158 had missing information about covariates), resulting in a study sample of 957 respondents in our final analyses. There was no significant difference in the outcome variables between individuals with complete and incomplete data (*P*>.05). The National Cancer Institute granted permission for the data used in this study.

### Measures

#### Independent Variable—Wearable Activity Tracker Use

Respondents were asked whether they have used electronic wearable activity trackers, such as a Fitbit, Apple Watch, or Garmin Vivofit, to monitor or track their health or activity in the past 12 months (yes=1, no=0).

#### Outcome Variables

Physical activity variables included the following two items: (1) time spent in moderate to vigorous physical activity, that respondents were asked how many minutes in a week they do any physical activity or exercise of at least moderate intensity such as brisk walking, bicycling, or swimming, and (2) weekly frequency of strength training, that the respondents were asked how many days they do physical activity or exercise specifically designed to strengthen their muscles such as lifting weights or circuit training. Health-related outcome variables included the following three items: (1) BMI, (2) anxiety and depression evaluated by the Patient Health Questionnaire-4 (ranged 0-12, a higher score means a higher level of anxiety and depression), and (3) self-rated health status (0=poor to 4=excellent).

#### Covariates

Sociodemographic characteristics included age (18-49, 50-64, 65-75, and 76 years or older), sex (male or female), race (White and non-White), education level (less than high school, high school, some college, or college graduate or higher), income, and employment status (employed or unemployed). Cancer-related factors included cancer types (breast, prostate, colorectal, skin, or other), stage (localized, regional, or distant), and time since diagnosis (<1 year, 2-5 years, 6-10 years, and 11 or more years). Other health-related factors included BMI (categorized as underweight, normal, overweight, or obese) and smoking status (current, former, or never). Of note is that we controlled for BMI in all other analyses, except for the linear regression, to examine BMI as the outcome variable. All data were self-reported.

#### Statistical Analysis

Descriptive statistics (mean, SE, and %) were calculated to describe wearable activity tracker use rate, physical activity, and health-related outcome variables, sociodemographic characteristics, and cancer- and health-related factors. Bivariate analyses were used to compare the sociodemographic and health-related characteristics of different groups (wearable activity tracker use or not); bivariate differences were assessed with Rao-Scott chi-square tests for categorical variables and design-based *F* tests for continuous variables. Design-based linear regression for continuous outcome variables and ordinal logistic regression for ordinal outcome variables were conducted to determine the associations after controlling for sociodemographic characteristics as well as cancer-related and general health–related clinical factors. For continuous outcome variables, the beta () coefficient was presented, while the odds ratio (OR) was provided for ordinal outcome variables. The findings were presented as weighted point estimates along with 95% CI. A significance level of .05 was used. The complex survey design and sample weights were considered in all analyses. Taylor series linearization methods were used for variance estimation. Stata/SE (version 17.0; StataCorp) was used to perform all the analyses.

### Ethical Considerations

The HINTS-SEER was approved as an amendment to HINTS 5 by the Westat institutional review board (IRB; amendment ID #3212), with participating SEER registries obtaining independent IRB approvals from 3 cancer registries, namely Iowa, New Mexico, and the Greater Bay Area [16]. The HINTS 5 was approved by the Westat IRB (project #6048.14) and received a “Not Human Subjects Research” determination from the National Institutes of Health Office of Human Subjects Research (Exempt #13204) [16]. Informed consent was obtained from all the participants [15]. Due to the use of a deidentified dataset, this study was exempted from undergoing an institutional ethical review [17]. This study has been conducted using the HINTS-SEER dataset with permission from the HINTS.

## Results

### Characteristics of Study Population

We included 957 participants, representing 320,164 patients with cancer. The majority were 65 years old or older (69%) and identified as White (78%). Over half of them were women (55%). Most had at least a college degree (62%), an annual income of US $50,000 or more (72%), and were not employed (70%). Breast cancer was the most prevalent (24%), followed by prostate (22%), skin (11%), and colorectal cancers (7%), with the majority being at a localized stage (70%). Over half of the patients had received a cancer diagnosis 11 or more years ago (58%). The majority of patients with cancer reported their health as good or better (86%), were overweight or obese (61%), and had never smoked (61%). The average anxiety and depression score was within the normal range (mean=1.54). Patients with cancer engaged in moderate to vigorous physical activity for an average of 171 minutes, and approximately 60% did not participate in any strength training. Only 29% of participants reported wearable activity tracker use. Bivariate analyses showed wearable activity tracker use was significantly associated with younger age (*P*<.001), higher education level (*P*=.04), higher income (*P*<.001), and employed status (*P*<.001); more details in Table 1.

**Table 1:** Characteristics of study population (design adjusted mean or proportion).

			Total		Wearable activity device use				*P* value^a^	
					No		Yes			
Sample size, N		957		—^b^		—		—		
National estimates^c^, %		—		70.89		29.11		—		
**Age (years), %**										<.001
	18-49	6.12		3.99		11.31				
	50-64	24.89		22.43		30.89				
	65-74	31.47		30.22		34.52				
	75+	37.52		43.36		23.28				
**Sex, %**										.18
	Male	45.08		46.64		41.27				
	Female	54.92		53.36		58.73				
**Race, %**										.93
	White	77.53		77.63		77.27				
	Non-White	22.47		22.37		22.73				
**Education level, %**										.04
	Less than high school	3.09		3.47		2.15				
	High school	10		11.49		6.38				
	Some college	25.24		27.09		20.76				
	College graduate or higher	61.67		57.95		70.71				
**Income (US $), %**										<.001
	<20,000	6.45		8.22		2.15				
	20,000-35,000	10.65		12.91		5.16				
	35,000-50,000	10.68		11.84		7.83				
	50,000-75,000	15.19		15.61		14.18				
	≥75,000	57.03		51.43		70.68				
**Employment, %**										<.001
	Employed	30.18		24.76		43.37				
	Unemployed	69.82		75.24		56.63				
**Cancer site, %**										.005
	Breast	24.46		25.10		22.89				
	Prostate	21.82		23.19		18.49				
	Colorectal	7.45		8.78		4.22				
	Skin	11.12		8.77		16.85				
	Other	35.14		34.16		37.54				
**Stage, %**										.81
	Localized	69.82		69.82		71				
	Regional	23.46		23.46		21.39				
	Distant	6.72		6.72		7.62				
**Time since diagnosis (years), %**										.26
	<1	0.92		1.02		0.95				
	2-5	16.65		14.74		16.65				
	6-10	24.63		24.98		24.63				
	11+	57.78		59.36		57.78				
**Smoking status, %**										.03
	Current	3.3		4.34		0.78				
	Former	36.19		37.06		34.06				
	Never	60.51		58.60		65.16				
**BMI, %**										.86
	Underweight	1.65		1.43		2.19				
	Normal	37.09		36.70		38.04				
	Overweight	34.53		34.77		33.96				
	Obese	26.73		27.10		25.81				
**Self-rated general health, %**										.002
	Excellent	9.11		7.58		12.85				
	Very good	35.85		32.99		42.84				
	Good	40.86		42.54		36.78				
	Fair	12.35		14.92		6.09				
	Poor	1.82		1.98		1.45				
Anxiety and depression, mean (SE)			1.54 (0.08)		1.59 (0.10)		1.43 (0.13)		.35	
Weekly minutes spent in moderate to vigorous physical activity, mean (SE)			171.08 (6.54)		157.34 (7.89)		204.54 (12.28)		.002	
**Weekly days spent in strength training, %**										<.001
	None	59.52		64.4		47.72				
	1 day	7.59		6.3		10.74				
	2 days	10.91		9.05		15.4				
	3 days	9.63		7.68		14.36				
	4 days	3.56		3		4.94				
	5 days	3.68		4.12		2.62				
	6 days	1.42		1.91		0.22				
	7 days	3.68		3.55		4.00				

^a^*P*-value compares older adults in different subgroups.

^b^Not available.

^c^National estimates based on complex survey design.

### Association Between Physical Activities and Wearable Activity Tracker Use

Table 2 illustrates the associations between 2 types of physical activities and wearable activity tracker use. The unadjusted regression analyses revealed a statistically significant association between wearable activity tracker use and higher weekly minutes spent in moderate to vigorous physical activity (=47.21, 95% CI 17.96-76.44, *P*=.002), as well as more weekly days spent in strength training (OR 1.67, 95% CI 1.24-2.24, *P*=.001). After accounting for covariates, these relationships remained statistically significant (Moderate to vigorous physical activity: =37.94, 95% CI 8.37-67.50, *P*=.01; Strength training: OR 1.49, 95% CI 1.08-2.06, *P*=.01). Among covariates, being female (= –61.30, 95% CI –98.14 to –24.45, *P*=.001), employed (=–36.33, 95% CI –67.87 to –4.80, *P*=.02), and obese (= –69.65, 95% CI –94.38 to –44.93, *P*<.001) were associated with decreased time in moderate to vigorous physical activity, while having an income of US $75,000 or higher (=72.29, 95% CI 19.28-125.31, *P*=.008) was associated with increased time in moderate to vigorous physical activity. Being White (OR 0.65, 95% CI 0.43-0.98, *P*=.04) and obese (OR 0.48, 95% CI 0.33-0.70, *P*<.001) were negatively associated with the frequency of strength training.

**Table 2:** Associations of wearable activity trackers used with physical activity (weighted estimates of adjusted coefficients and adjusted odds ratios [ORs]).

				Time spent in moderate to vigorous physical activity^a^				Frequency of strength training^b^		
				Adjusted OR	95% CI	*P* value	Adjusted OR		95% CI	*P* value
Wearable activity trackers use (reference: no use)				37.94	8.37-67.50	.01	1.49		1.08-2.06	.01
**Age (years)**										
	18-49			Reference	—^c^	—	Reference		—	—
	50-64			47.13	–11.62 to 105.90	.12	1.31		0.63-2.71	.45
	65-74			57.70	–2.44 to 117.85	.06	1.42		0.68-2.98	.34
	75+			45.65	–17.53 to 108.83	.16	0.94		0.42-2.09	.89
**Sex**										
	Male			Reference	—	—	Reference		—	—
	Female			–61.30	–98.14 to –24.45	.001	0.71		0.48-1.05	.08
**Race**										
	Non-White			Reference	—	—	Reference		—	—
	White			10.78	–18.37 to 39.93	.46	0.65		0.43-0.98	.04
**Educational level**										
		Less than high school		Reference	—	—	Reference		—	—
		High school		42.45	–28.49 to 113.40	.24	1.45		0.55-3.79	.44
		Some college		10.97	–52.30 to 74.26	.73	1.12		0.48-2.58	.78
		College graduate or higher		44.40	–15.24 to 104.06	.14	1.46		0.65-3.27	.34
**Income (US $)**										
		<20,000		Reference	—	—	Reference		—	—
		20,000-35,000		–44.00	–94.55 to 6.53	.08	1.06		0.46-2.47	.87
		35,000-50,000		19.75	–35.24 to 74.75	.48	1.91		0.84-4.35	.12
		50,000-75,000		31.83	–24.07 to 87.74	.26	1.16		0.50-2.66	.71
		≥75,000		72.29	19.28 to 125.31	.01	1.46		0.71-2.98	.29
**Employment**										
			Unemployed	Reference	—	—	Reference		—	—
		Employed		–36.33	–67.87 to –4.80	.02	1.43		0.99-2.08	.05
**Cancer site**										
	Breast			Reference	—	—	Reference		—	—
	Prostate			–25.58	–77.78 to 26.61	.33	1.34		0.71-2.51	.35
	Colorectal			–5.46	–60.57 to 49.65	.84	1.19		0.59-2.36	.61
	Skin			–0.18	–38.05 to 37.69	.99	1.38		0.80-2.35	.23
	Other			–26.73	–56.93 to 3.46	.08	1.04		0.64-1.68	.86
**Cancer stage**										
	Localized			Reference	—	—	Reference		—	—
	Regional			—	–15.93 to 44.00	.35	1.06		0.69-1.63	.77
	Distant			—	–71.60 to 29.13	.41	1.03		0.52-2.03	.92
**Time since diagnosis (years)**										
	1			Reference	—	—	Reference		—	—
	2-5			84.30	–0.20 to 168.80	.05	0.99		0.33-2.93	.99
	6-10			64.84	–13.98 to 143.67	.11	1.50		0.56-4.03	.41
	11+			68.78	–11.51 to 149.09	.09	1.38		0.49-3.86	.52
**BMI**										
	Underweight			—	–131.75 to 145.42	.92	1.30		0.60-2.85	.49
	Normal			Reference	—	—	Reference		—	—
	Overweight			—	–36.25 to 30.59	.86	0.69		0.48-1.00	.05
	Obese			—	–94.38 to –44.93	<.001	0.48		0.33-0.70	<.001
**Smoking status**										
	Current			Reference	—	—	Reference		—	—
	Former			–21.83	–95.41 to 51.74	.55	0.73		0.31-1.68	.46
	Never			7.53	–67.96 to 83.03	.84	0.84		0.35-1.96	.68

^a^Design-based linear regression, the residuals were close to a normal distribution.

^b^Design-based ordinal logistic regression.

^c^Not applicable.

### Association Between Health Outcomes and Wearable Activity Tracker Use

The unadjusted regression analyses revealed a statistically significant association between wearable activity tracker use and better self-rated health status (OR 1.91, 95% CI 1.38-2.63, *P*<.001), and this association remained statistically significant after controlling for covariates (OR 1.58, 95% CI 1.09-2.28, *P*=.01; more details in Table 3). However, BMI and anxiety and depression were not significantly associated with wearable activity tracker use (more details in Multimedia Appendix 1).

**Table 3:** Associations of wearable activity trackers used with self-rated health status (weighted estimates of adjusted coefficients and adjusted odds ratios [ORs]).

		Self-rated general health^a^			Anxiety and depression^b^		
		Adjusted OR	95% CI	*P* value	Adjusted OR	95% CI	*P* value
Wearable activity device use (reference: no use)		1.58	1.09-2.28	.01	–0.16	–0.48 to 0.17	.35
**Age (years)**							
	18-49	Reference	—^c^	—	Reference	—	—
	50-64	0.97	0.46-2.03	.94	–0.34	–1.09 to 0.41	.37
	65-74	0.95	0.45-1.99	.90	–0.48	–0.31 to 0.35	.26
	75+	1.01	0.47-2.13	.98	–0.56	–1.48 to 0.36	.23
**Sex**							
	Male	Reference	—	—	Reference	—	—
	Female	0.89	0.57-1.41	.63	0.56	0.16-0.97	.01
**Race**							
	Non-White	Reference	—	—	Reference	—	—
	White	1.26	0.83-1.92	.26	–0.08	–0.57 to 0.42	.76
**Educational level**							
	Less than high school	Reference	—	—	Reference	—	—
	High school	1.41	0.66-3.04	.36	0.65	–0.31 to 1.61	.18
	Some college	1.60	0.75-3.38	.21	0.40	–0.47 to 1.26	.37
	College graduate or higher	2.54	1.20-5.36	.01	0.01	–0.82 to 0.85	.98
**Income (US $)**							
	<20,000	Reference	—	—	Reference	—	—
	20,000-35,000	0.96	0.46-2.03	.93	0.92	0.23-1.61	.01
	35,000-50,000	2.58	1.27-5.23	.01	0.35	–0.50 to 1.20	.41
	50,000-75,000	2.44	1.18-5.04	.01	0.04	–0.56 to 0.65	.88
	≥75,000	2.23	1.14-4.33	.01	0.08	–0.47 to 0.62	.78
**Employment**							
	Unemployed	Reference	—	—	Reference	—	—
	Employed	1.39	0.96-2.01	.07	–0.09	–0.54 to 0.36	.70
**Cancer site**							
	Breast	Reference	—	—	Reference	—	—
	Prostate	1.59	0.87-2.90	.12	0.11	–0.59 to 0.81	.75
	Colorectal	1.06	0.57-1.95	.84	–0.65	–1.23 to –0.07	.03
	Skin	1.83	1.10-3.05	.02	0.51	–0.15 to 1.18	.13
	Other	0.88	0.57-1.37	.58	–0.08	–0.56 to 0.40	.76
**Cancer stage**							
	Localized	Reference	—	—	Reference	—	—
	Regional	1.14	0.76-1.71	.50	–0.10	–0.48 to 0.28	.61
	Distant	0.79	0.39-1.58	.50	0.14	–0.66 to 0.95	.73
**Time since diagnosis (years)**							
	1	Reference	—	—	Reference	—	—
	2-5	2.29	0.64-8.14	.19	–0.93	–3.05 to 1.20	.39
	6-10	2.12	0.66-6.82	.20	–0.67	–2.74 to 1.41	.53
	11+	2.07	0.61-6.98	.23	–1.05	–3.09 to 0.99	.31
**BMI**							
	Underweight	0.31	0.12-0.80	.01	1.03	–0.13 to 2.20	.08
	Normal	Reference	—	—	Reference	—	—
	Overweight	0.58	0.42-0.80	.001	0.24	–0.15 to 0.62	.22
	Obese	0.31	0.22-0.44	<.001	0.02	–0.44 to 0.47	.95
**Smoking status**							
	Current	Reference	—	—	Reference	—	—
	Former	1.69	0.66-4.31	.26	–0.64	–1.66 to 0.39	.22
	Never	1.69	0.70-4.04	.23	–0.72	–1.65 to 0.21	.13

^a^Design-based ordinal logistic regression.

^b^Design-based linear regression, the residuals were close to a normal distribution.

^c^Not applicable.

## Discussion

### Principal Findings

This study used a national dataset to examine the association between using wearable activity trackers and physical activity and health outcomes among patients with cancer. The key findings were that (1) overall wearable activity tracker use was low (29%), and patients with no wearable activity tracker use tended to be older and of low socioeconomic status; (2) using wearable activity trackers was associated with increased weekly duration of moderate to vigorous physical activity, weekly frequency of strength training and improved self-rated health. Associations were not observed between using wearable activity trackers and BMI or anxiety and depression. Based on the nature of this cross-sectional study, we could not draw a causality of the benefits of using wearable activity trackers. However, the findings still advance our understanding of the use of wearable activity trackers and support using wearable activity trackers as a promising behavior modification tool to facilitate physical activity promotion.

This study showed the association between wearable activity tracker use and physical activity level (aerobic and strength) among patients with cancer. This is consistent with observational studies of general adults [12] and older adults [13] and meta-analyses of clinical trials [8,10,18]. These trackers provide real-time feedback on physical activity levels and allow users to self-monitor and set goals [10]; patients may perceive the devices as a reminder of physical activity, and they are motivated to do more physical activity when feeling they are observed [19]. A review of systematic review has reported an effect size of 0.3 for wearable activity trackers on moderate to vigorous physical activity in US adults, equating to a 6-minute-per-day increase in moderate to vigorous physical activity [8]. Interestingly, our study has found a similar result (an increase of 5.4 minutes per day) among patients with cancer. An increase of 5-10 minutes of moderate to vigorous physical activity per day is considered meaningful [9], suggesting the magnitude of moderate to vigorous physical activity increase is clinically significant. The evidence supports the use of wearable activity trackers to facilitate physical activity promotion.

Our study found an association between wearable activity trackers and self-rated health, which is consistent with another study using the HINTS dataset [11]. The association of wearable activity trackers and self-rated health is mediated through perceived health competence and physical activity; an approach to foster the perceived health competence includes setting challenges and meaningful feedback by wearable activity trackers [11]. We did not detect the association between wearable activity trackers and other physiological and psychosocial outcomes. One possible explanation is that these outcomes are probably downstream of physical activity behavior change and might require a long duration of wearable activity trackers use to manifest.

We found the prevalence of wearable activity tracker use among patients with cancer was low (29%), which is similar to the estimates of adults in the United States [12], although higher than the older adults [13]. Our study also found that patients with no wearable activity tracker use tended to be older, unemployed, and with lower income, highlighting the digital divide and health inequity. As the study findings support the positive association between wearable activity tracker use and improved physical activity and health status, this study’s findings can inform researchers on how to mitigate the digital divide and promote digital health literacy [20] to facilitate wearable activity tracker use in patients with cancer. Wide reach to patients of older age and low socioeconomic status is key to guaranteeing that physical activity promotion using wearable activity trackers generates large effects on a broad population without worsening health inequities.

### Limitations

While this study used a population-based dataset acquired from 3 prominent cancer registries in the United States, the geographical aspects involved could potentially affect the generalizability of the study findings. Also, the cross-sectional analysis prevents us from drawing solid conclusions about the direction of the relationships between wearable activity trackers and physical activity and health outcomes. Future longitudinal research addressing this research gap would be beneficial. In addition, the self-reported measures of physical activity are not as accurate as objectively measured physical activity, and the details about the wearable activity tracker use (eg, wear time, type of wearable activity trackers, etc) were not available in the HINTS-SEER dataset, which may moderate the relationship between wearable activity tracker use and physical activity. We only included several health outcome indicators and other important health outcomes (eg, quality of life, fatigue) were not available for analysis.

### Conclusion

This research investigated the associations between the use of wearable activity trackers, physical activity, and health outcomes in patients with cancer using a population-based dataset. The results of the study indicate that the adoption of wearable activity trackers among patients with cancer remains relatively low. However, the findings also provide evidence that using wearable activity trackers is associated with increased physical activity and improved overall health, thus suggesting their potential as effective tools for promoting physical activity. Nonetheless, the study revealed a lower usage rate of wearable activity trackers among older individuals and those with a lower socioeconomic status, underscoring the presence of a digital divide within the population of patients with cancer. Further research is necessary to explore strategies for increasing the use of wearable activity trackers in cancer survivors who are older and have lower socioeconomic status.

## Appendix

Multimedia Appendix 1 Weighted estimates of adjusted coefficients in linear regression models of BMI and anxiety/depression.

## Abbreviations

HINTS: Health Information National Trends Survey
HINTS-SEER: Health Information National Trends Survey–Surveillance, Epidemiology, and End Results
IRB: institutional review board
OR: odds ratio
