# Supplementary material for: Associations of Wearable Activity Tracker Use With Physical Activity and Health Outcomes in Patients With Cancer: Findings from a Population-Based Survey Study
Source: J Med Internet Res. 2024 Oct 22;26:e51291. doi: 10.2196/51291 (PMC11538876; doi:10.2196/51291)
Supplement: Multimedia Appendix 1 [file jmir_v26i1e51291_app1.docx]

Multimedia Appendix 1. Weighted estimates of adjusted coefficients in linear regression models of BMI

|  | BMI | | | |
| --- | --- | --- | --- | --- |
|  | Adjusted β | 95% CI | | *P* |
| Wearable activity device use | -0.19 | -1.27 | 0.90 | 0.74 |
| Age |  |  |  |  |
| 18-49 | Ref |  |  |  |
| 50-64 | 1.86 | -0.29 | 4.01 | 0.09 |
| 65-74 | 1.12 | -1.36 | 3.60 | 0.37 |
| 75+ | -1.02 | -3.54 | 1.50 | 0.43 |
| Sex |  |  |  |  |
| Male | Ref |  |  |  |
| Female | 0.91 | -0.32 | 2.15 | 0.15 |
| Race |  |  |  |  |
| Non-White | Ref |  |  |  |
| White | 0.18 | -1.09 | 1.45 | 0.78 |
| Educational Level |  |  |  |  |
| Less than high school | Ref |  |  |  |
| High school | -0.92 | -4.74 | 2.91 | 0.64 |
| Some college | -0.58 | -4.23 | 3.07 | 0.76 |
| College graduate or higher | -2.79 | -6.37 | 0.80 | 0.13 |
| Income |  |  |  |  |
| Less than 20k | Ref |  |  |  |
| 20k to <35k | 1.05 | -1.13 | 3.24 | 0.34 |
| 35k to <50k | 0.70 | -1.32 | 2.73 | 0.49 |
| 50k to <75k | 0.46 | -1.60 | 2.53 | 0.66 |
| 75k or more | -0.81 | -2.68 | 1.05 | 0.39 |
| Employment |  |  |  |  |
| Unemployed | Ref |  |  |  |
| Employed | 0.09 | -1.36 | 1.55 | 0.90 |
| Cancer site |  |  |  |  |
| Breast | Ref |  |  |  |
| Prostate | 1.19 | -0.52 | 2.90 | 0.17 |
| Colorectal | -0.24 | -1.72 | 1.24 | 0.75 |
| Skin | -0.07 | -1.62 | 1.48 | 0.93 |
| Other | 2.13 | 0.85 | 3.40 | 0.00 |
| Cancer stage |  |  |  |  |
| Localized | Ref |  |  |  |
| Regional | -0.45 | -1.49 | 0.60 | 0.40 |
| Distant | -1.15 | -3.36 | 1.06 | 0.30 |
| Time since diagnosis |  |  |  |  |
| ≤ 1 year | Ref |  |  |  |
| 2-5 years | 2.55 | 0.59 | 4.50 | 0.01 |
| 6-10 years | 3.28 | 1.22 | 5.34 | 0.00 |
| 11+ years | 2.37 | 0.52 | 4.21 | 0.01 |
| Smoking status |  |  |  |  |
| Current | Ref |  |  |  |
| Former | 2.78 | 0.28 | 5.29 | 0.03 |
| Never | 2.55 | 0.19 | 4.91 | 0.03 |

Note. The categorical variable of BMI was not controlled for in this linear regression.
